# A review of the extraction and purification methods, biological activities, and applications of active compounds in *Acanthopanax senticosus*

**DOI:** 10.3389/fnut.2024.1391601

**Published:** 2024-05-23

**Authors:** Xindi Zhang, Lijun Guan, Ling Zhu, Kunlun Wang, Yang Gao, Jialei Li, Song Yan, Nina Ji, Ye Zhou, Xinmiao Yao, Bo Li

**Affiliations:** ^1^Food Processing Institute, Heilongjiang Academy of Agricultural Sciences, Harbin, China; ^2^Key Laboratory of Food Processing of Heilongjiang Province, Harbin, China; ^3^Soybean Institute, Heilongjiang Academy of Agricultural Sciences, Harbin, China

**Keywords:** *Acanthopanax senticosus*, eleutheroside, polysaccharide, flavonoid, biological activity, application

## Abstract

*Acanthopanax senticosus* (AS) is a geo-authentic crude medicinal plant that grows in China, Korea, Russia, and Japan. AS contains bioactive compounds such as eleutherosides, polysaccharides, and flavonoids. It is also a key traditional herb in the Red List of Chinese Species. AS is mainly distributed in Northeast China, specifically in Heilongjiang, Jilin, and Liaoning provinces. Its active compounds contribute to significant biological activities, including neuroprotective, antioxidant, anti-fatigue, and antitumor effects. However, the extraction methods of active compounds are complex, the extraction efficiency is poor, and the structure–activity relationship is unclear. This study focused on the nutrients in AS, including protein, carbohydrates, and lipids. Particularly, the active ingredients (eleutherosides, polysaccharides, and flavonoids) in AS and their extraction and purification methods were analyzed and summarized. The biological activities of extracts have been reviewed, and the mechanisms of anti-oxidation, antitumor, anti-inflammation, and other activities are introduced in detail. The applications of AS in various domains, such as health foods, medicines, and animal dietary supplements, are then reported. Compared with other extraction methods, ultrasonic or microwave extraction improves efficiency, yet they can damage structures. Challenges arise in the recovery of solvents and in achieving extraction efficiency when using green solvents, such as deep eutectic solvents. Improvements can be made by combining extraction methods and controlling conditions (power, temperature, and time). Bioactive molecules and related activities are exposited clearly. The applications of AS have not been widely popularized, and the corresponding functions require further development.

## 1 Introduction

**Figure 1:**
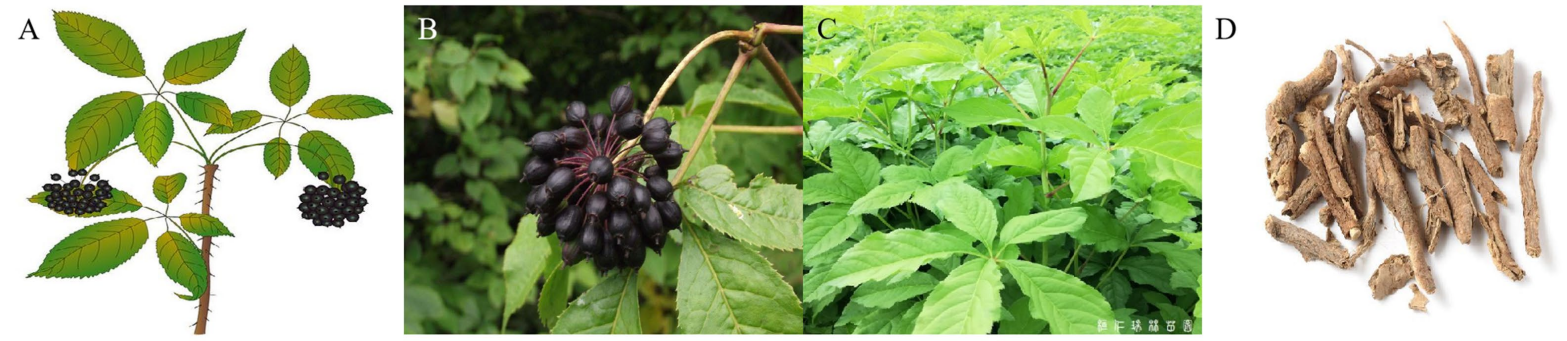
The **(A)** overview, **(B)** fruits, **(C)** leaves, and **(D)** barks of *Acanthopanax senticosus*.

*Acanthopanax senticosus* (AS), commonly known as “Ci-wu-jia” in China, originates from the dried root and rhizome or stem of AS (Rupr. et Maxim.) and belongs to the Araliaceae family (1). AS is a shrub that grows in forests or shrub areas, typically ranging from 1 to 4 m in height (Figure 1). It is also known as “Siberian ginseng” and is found across China, South Korea, Japan, and Russia (2). In China, it mainly grows in Northeastern provinces, such as Xiaoxing’anling in Heilongjiang, the Changbai Mountains in Jilin, Shenyang in Liaoning, and Hebei and also Shanxi provinces in North China.

The medicinal effect of AS, which has a bitter and astringent taste, is mild. It is considered to nourish the spleen and stomach and stimulate appetite (3). Its active ingredients include glycosides, polysaccharides, flavonoids, lignans, triterpenoids, and organic acids (4). Modern pharmacology has revealed that AS can regulate immunity, treat cardiovascular system diseases, and has anti-inflammatory, antitumor, and anti-oxidation effects (5). Glycosides repel insects, have antibacterial properties, and can improve sleep (6). Polysaccharides and their derivatives considerably affect the treatment of malignant tumors and improve human immunity due to their unique biological activities (7). Moreover, flavonoids have been reported to slow down human aging (3). Previous research involving a systematic study on Araliaceae plants has shown that AS and ginseng have similar pharmacological effects and clinical efficacy (8).

In terms of food supplements, the tender stems and fresh leaves of AS contain carotene, riboflavin, ascorbic acid, and rich vitamins. Its stem flavor is unique, aromatic, and slightly bitter. AS can be used as an ingredient in cooking and soups, and it can also be used to make packaging cans. Its leaves can be processed to produce a light-brown tea with a distinctive aroma and sweet taste.

**Figure 2:**
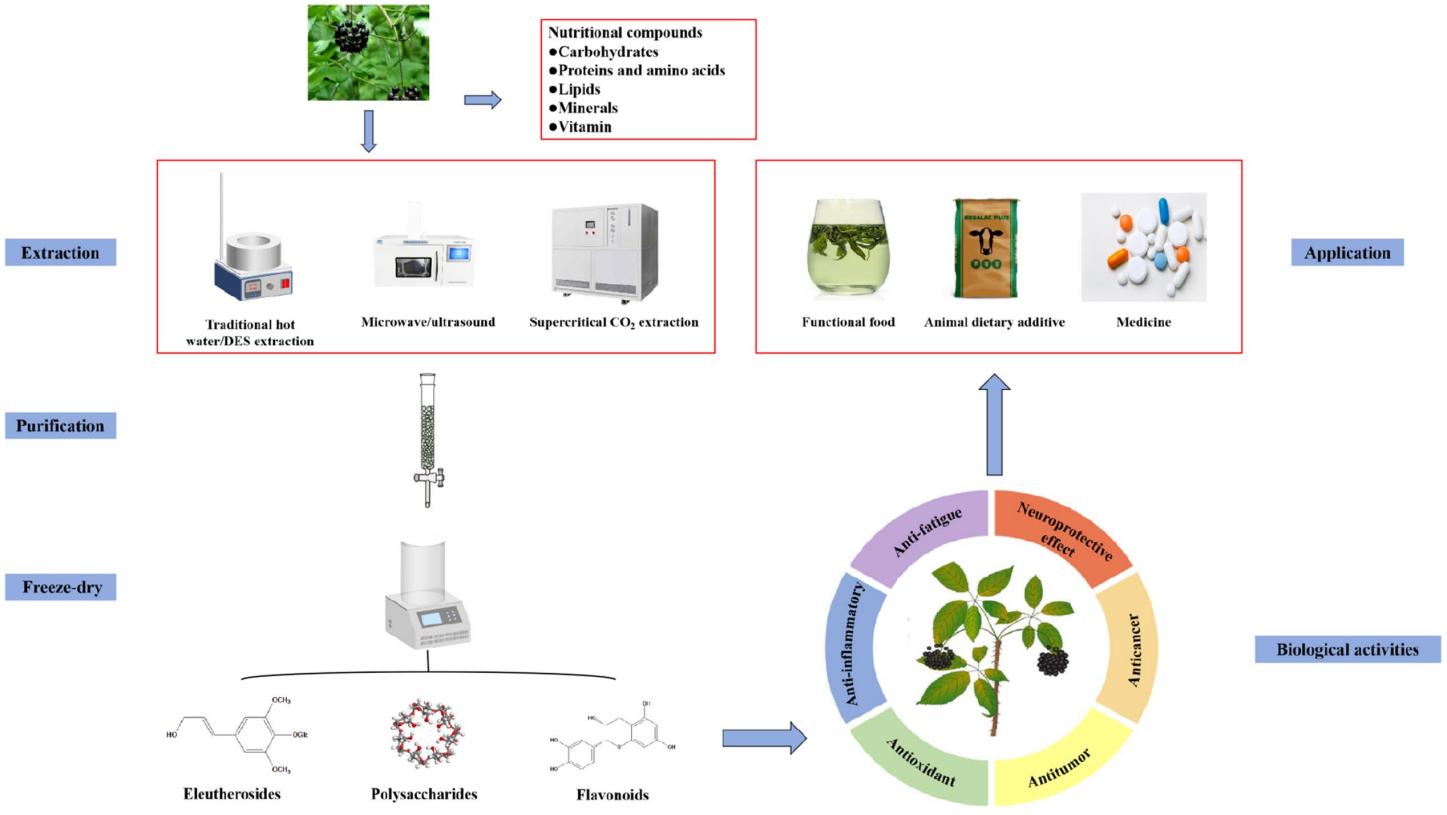
Schematic diagram of extraction, purification, biological activities, and application of *Acanthopanax senticosus*.

In this study, the nutritional components, active components, extraction and purification methods, biological activities, and applications of AS are presented and discussed to expand its application range. Figure 2 presents a schematic diagram of the study structure.

## 2 Nutritional compounds

AS contains numerous nutrients, such as polysaccharides, essential amino acids, lipids, and vitamins, which are necessary for humans in regulating metabolism (Table 1). Table 2 summarizes the benefits of AS for humans in different countries. The results showed that there was a dose-dependent relationship between carbohydrate accumulation and the nitrogen application rate in AS (19). Furthermore, this content is related to environmental factors such as soil, altitude, moisture, sunshine, and topography (20). Processes such as decoction and fermentation increase the content of soluble solids and polysaccharides (21).

**Table 1:** Contents of nutritional compounds (%) in *Acanthopanax senticosus* of different habitats.

Compounds			Content (%)	Habitats	References
Moisture			7.50–87.9 (fresh weight)	Heilongjiang, China Autonomous, Yunnan, China	(9, 10)
Carbohydrate			72.33–89.47	Chuncheon, South Korea	(11)
	Monosaccharides			Liaocheng, China	(12)
		Glucose	6.05%		
		Arabinose	13.48%		
		Xylose	0.72%		
		Mannose	2.02%		
		Galactose	5.40%		
		Rhamnose	18.22%		
	Disaccharides			Heilongjiang, China	(13)
		β-Gentiobiose	-		
		D-Cellobiose	-		
	Polysaccharide		4.36%	Heilongjiang, China	(14)
	Reducing sugar		6.24–20.80	Heilongjiang, China	(9)
	Crude fiber		2.21–5.74%	Jilin, China	(15)
Protein			2.08–2.74	Liaocheng, China	(16, 17)
	Free amino acid				
		Alanine	0.12–0.64		
		Lysine	0.17–1.62		
		Aspartate	0.23–1.48		
		Glutamate	0.26–2.06		
		Valine	0.23–0.62		
		Isoleucine	0.12–0.63		
		Phenylalanine	0.13–0.61		
		Leucine	0.21–0.94		
		Threonine	0.08–0.14		
Lipid			-	Jilin, China Seoul, Korea	(18)
	Unsaturated fatty acid				
		Methyl oleate	-		
		Ethyl oleate	-		
		Methyl 10, 13-octadecadienoate	-		
		Ethyl 10, 13-octadecadienoate	-		
	Saturated fatty acid	Myristic acid	-		
Mineral				Jilin, China	(10)
	K		(0.62–480) × 10^−3^		
	Ca		(0.60–90) × 10^−3^		
	Na		(5.28–1,000) × 10^−6^		
	Mg		(0.11–50) × 10^−3^		
	Fe		(0.12–1) × 10^−3^		
	Cu		(0.02–0.2) × 10^−3^		
	Zn		(0.05–0.6) × 10^−3^		
	Mn		10^−3^		
	Se		0.4 × 10^−3^		
Vitamin	V_C_		(7–11, 13, 15, 19–21) ×10^−3^	Heilongjiang, China	(22, 23)
	V_B1_		0.96 × 10^−6^		
	V_B2_		0.05 × 10^−6^		
	V_B3_		0.67 × 10^−6^		
	V_B6_		2.16 × 10^−6^		
	V_B12_		0.02 × 10^−6^		

**Table 2:** The health-promoting effects of *Acanthopanax senticosus* from different countries.

Health-promoting benefits	Part/ingredient	*In vitro* or *in vivo*	Research outcome	Country	References
Antiaging/hepatoprotective	Stem, root/extract	*In vivo*	AS attenuated leukocyte telomere length shortening and reduce liver biochemical parameters	Korea	(24)
Immunomodulatory effect	Stems, leaves/polysaccharide	*In vivo*	AS promoted lymphocyte proliferation, and resist immunosuppression induced by cyclophosphamide	China	(25)
Treat type 2 diabetes	Stem/polysaccharide	*In vivo*	AS improved glucose tolerance and alleviate insulin resistance	China	(26)
Nerve protection-learning and memory impairment	N.D./polysaccharide	*In vivo*	AS improved learning and memory ability in mice and inhibited inflammation levels	China	(27)
Nerve protection-PD	Extract/N.D.	*In vivo*	AS increased the number of autonomous movements and impacted a key metabolic pathway of PD by regulating gut microbial structure and metabolic disorders	China	(28)
Nerve protection-PD	Root/extract	*In vivo*	Protein expression changed significantly after AS treatment	China	(29)
Nerve protection-Alzheimer’s disease	N.D.	Molecular docking	Eleutheroside B, chiisanoside, and eleutheroside D_1_ had strong binding abilities to key target proteins	China	(30)
Treat liver injury/antioxidant	Decoction pieces	*In vivo*	AS regulated antioxidant and antiapoptotic-related gene expression levels	China	(31)
Anti-depression	N.D.	*In vivo* and *in vitro*	AS improved depression and simultaneously ameliorate hepatic metabolomic alterations in Chronic unpredictable mild stress mice	China	(32)
Treat radiation-induced brain injury	Roots, leaves/polysaccharide	*In vivo*	AS protected the neurons of irradiated mice and prevent the irradiated mice from learning and memory ability impairment	China	(33)
Anti-atherosclerotic	N.D.	*In vivo*	AS regulated via the nuclear factor-kappa pathway	China	(34)
Immunomodulatory effect	Nanoemulsion/polysaccharide	*In vivo*	AS induced several cytokines (IL-2, IL-6, TNF-α, and IFN-γ) involved in the TLR4-NF-κB signal transduction pathway-induced immunoregulation signaling	China	(35)

^a^“N.D.” means “not detected”.

### 2.1 Carbohydrates

The carbohydrates in AS mainly consist of monosaccharides, disaccharides, polysaccharides, reducing sugar, and crude fiber. Carbohydrates accounted for 72.33% of the total composition in AS (11). The major monosaccharides in AS leaves are glucose, xylose, and rhamnose. Reduced sugar and polysaccharides in AS leaves are more accumulated during the growth and deciduous periods compared to other periods (13). The content of reduced sugar and polysaccharides is approximately 6.24–20.80% (9) and 2.21–5.74% (15), respectively, of the total composition in AS.

### 2.2 Proteins and amino acids

The protein content in AS ranges from 2.08 to 2.74% (9). The category, content, and ratio of essential amino acids are important indicators for evaluating plant nutrition (10). Su et al. found that the content of amino acids was the highest in seeds. Moreover, amino acids in AS leaves accumulate more during the flourishing and declining stages of growth than in other periods (13). AS consists of amino acid varieties, including alanine, glutamic acid, and aspartic acid. Researchers have identified approximately 13 different amino acids (6 essential amino acids) in AS, with 4 major acids such as glutamic (11.40–15.54% of the total amino acids), aspartic acid (10.08–11.16%), and lysine acids (7.46–12.22%) (10).

### 2.3 Lipids

Lipids are the main components of plant cell organ membranes and provide energy for metabolism. The fatty acids of leaves accumulate in the growth period to obtain energy (13). Crude fat compromises 2.07% ± 0.09% of fatty acids in AS, including 12.98% saturated fatty acids, 33.13% unsaturated fatty acids, 27.46% unsaturated alcohols, and 15.76% diolefins (9). Zhao et al. isolated and characterized eight fatty acids from the AS root bark extract, including oleic acid methyl acetate, oleic acid ethyl acetate, 10,13-octadecadienoic acid methyl acetate, 10,13-octadecadienoic acid ethyl acetate, myristic acid, palmitoleic acid, 9, 11-octadecadienoic acid, and hexadecatrienoic acid (18).

### 2.4 Minerals

The mineral content in AS varies with the species, origin, and climate. Previous research has identified the major and trace elements of AS (10), including minerals such as potassium, calcium, sodium, magnesium, iron, copper, zinc, manganese, and selenium.

### 2.5 Vitamins

AS is rich in vitamins such as V_B1_, V_B2_, V_B3_, V_B6_, V_B12_, and V_C_ (9), providing the ability to stimulate cells and enhance immunity.

## 3 Active compounds

### 3.1 Polysaccharides

Polysaccharides are polymers linked by multiple glycosidic bonds. The structural analysis of polysaccharides focuses on their primary structure, such as monosaccharide composition, relative molecular mass, and chain conformation analysis. Understanding the structural features of polysaccharides is essential for assessing their functions in biological systems and their macroscopic properties in industrial applications.

The hydrolysis of polysaccharides is typically performed using trifluoroacetic acid (TFA) or specific enzymes to treat the polysaccharide chain and obtain the main chain and fragment structure. These are then combined with techniques such as monosaccharide composition, methylation, and nuclear magnetic resonance for the structure resolution of degraded products (36). Chen et al. extracted crude polysaccharides with antioxidant and immunological activities and subsequently hydrolyzed them with TFA to obtain water-soluble polysaccharides with a relative molecular mass of 14.57 kDa (12). As shown in Table 3, the molecular weights of the polysaccharides of AS range between 5.24 and 169 kDa. The diversities in the molecular weights may be due to the differences in the sources of raw materials, extraction temperatures, and deproteinization methods. The structure and type of polysaccharides in the stem of AS are more complex than those in the leaves, and the fibrous structure of the stem protects the plant cells from damage for better bud differentiation. The high temperature during extraction and the use of dialysis bags during deproteinization will destroy the structure of polysaccharides.

**Table 3:** Summary of the extraction and purification process of polysaccharides from *Acanthopanax senticosus*.

		Extraction					Purification					
Origin	Part	Solvent	S/L ratio	Power (W)	Temperature (°C)	Time (h)	Precipitation	Removal of protein	Content (%)	Molecular weight (kDa)	Structure	References
Shanxi, China	Root, stem	Water	1: 50	–	–	1	95% ethanol	–	1.68	–	–	(37)
Zhejiang, China	–	Alkali-extraction	1: 24	–	–	–	60% ethanol	CHCl_3_/C_4_H_9_OH: 4: 1	6.01	–	Arabinose:xylose: galactose:glucose = 14:34:34:18	(38)
		Water			–				5.19	–	Arabinose:xylose: galactose:glucose = 12:45:33:11	
–	Root	Water	1: 20	–	–	1.5	–	–	10.14	–	–	(39)
Hunan, China	Leaf	Water	1: 14	–	90	6	80% ethanol	CHCl_3_/C_4_H_9_OH:=3: 1, DEAE-sepharose Fast Flow and dextran gel G-75column chromatography	1.94		Galactose, rhamnose, glucose	(40)
Jilin, China	Stem	Water	–	–	–	–	–	Ion exchange chromatographic columns DEAE-52	ASP-I: 87.61, ASP-II: 72.33		ASP-I: Mannose:glucose:galactose:arabinose =1:6.63:2.7:0.88 ASP-II: Mannose:rhamnose:galacturonic acid:glucose:galactose:arabinose = 1:2.28:4.30:1.18:2.82:2.81	(41)
Gansu, China	Fruit	Water	1: 15	Ultrasound: 325	47	0.4	85% ethanol	CHCl_3_/C_4_H_9_OH: 4: 1	3.81	11.2–133.5	Glucose:galactose:rhamnose:arabinose:mannose:xylose:glucuronic =8.83:7.90:4.74:4.55:2.80:2.39:1.00	(42)
Gansu, China	Stem	Water	1: 30	–	60	15	90% ethanol	CHCl_3_/C_4_H_9_OH: 4: 1	1.5	169	Galactose:glucose:mannose:arabinose = 6.1:2.1:1.1:1.0	(43)
–	Bark	Water	1: 25	Ultrasound: 85	58	1.2	Water	Dialysis bag: cut-off molecular weight (MW) = 7,000 Da	1.53		–	(44)
Heilongjiang, China	Stem, fruit	Water	1: 50	Ultrasound:100	80	1.25	Water	Dialysis bag: cut-off MW = 3,500 Da	1.1	–	–	(45)
Jilin, China	Stem	Water	–	–	–	0.2	80% ethanol	DEAE-52 Cellulose Ion Exchange Columns and dextran gel G-75 column chromatography	-		Mannose, glucose, galactose, arabinose	(46)
-	Leaf	Water		–	90	4	70% ethanol		5.2	14.57	Molar ratio: rhamnose, xylose, glucose, mannose, arabinose, galactose and glucuronic acid = 7.45:18.63:25.15:0.93:8.35:2.79:5.69	(12)
Sichuan, China	Fruit	Water	1: 10	–	100	5	Ethanol	–	1.05	AHP-I: 6.2	Arabinose, fucose, mannose, glucose, galactose = 1.00:1.40:0.64:3.13:2.09	(47)
										AHP-II: 64.4	Rhamnose, arabose, mannose, glucose and galactose = 0.36:1.00:0.15:0.20:1.10	
										AHP-III: 12.1	Rhamnose, arabinose, fucose, mannose, glucose and galactose = 1.22:1.00:0.40:0.28:0.26:1.03	
Yamagata Prefecture, Japan	Leaf	95% ethanol	1: 1	–	25	16	–	–	3.25	ANP: 10.7	L-arabinose, D-mannose, D-glucose and D-galactose 1.0:2.6:2.5:1.4	(48)
										AAP: 84	Larabinose, D-galactose, 4-O-methyl-d-glucuronic acid = 5:10:1	
Gansu, China	Stem, bark	Water	1: 30	–	80	10	50% ethanol	–	1.70	106	Galactose, glucose, rhamnose and galacturonic acid = 3.0:2.0:2.0:1.0	(49)
Heilongjiang, China	Leaf	Water	1: 2	-	90	3	80% ethanol	Dialysis bag: cut-off MW = 3,500 Da	1.67	ASPB_2_: 5.24	GalA, Ara, Rha, Man, Glc and Gal = 59.9:2.6:8.6:6.4:6.5:16.3	(50)
										ASPB_3_: 30.51 kDa	GalA, Ara, Rha, Man, Glc and Gal = 42.5:2.5:7.6:14.5:15.2:17.7	

Structural characterization of polysaccharides isolated from the different parts of *Acanthopanax senticosus*.

### 3.2 Eleutherosides

Numerous studies have identified eleutherosides as the main components of AS with pharmacological effects. AS contains 16 types of eleutherosides, compromising 18.59% of the total content, in its different components (roots, stems, leaves, etc.). The type and content vary with the plant component. In addition, the eleutheroside content can also vary within the same plant component. Scholars have determined the content of eleutherosides in the roots, stems, and leaves as 1.05, 2.49, and 0.75%, respectively (51).

**Figure 3:**
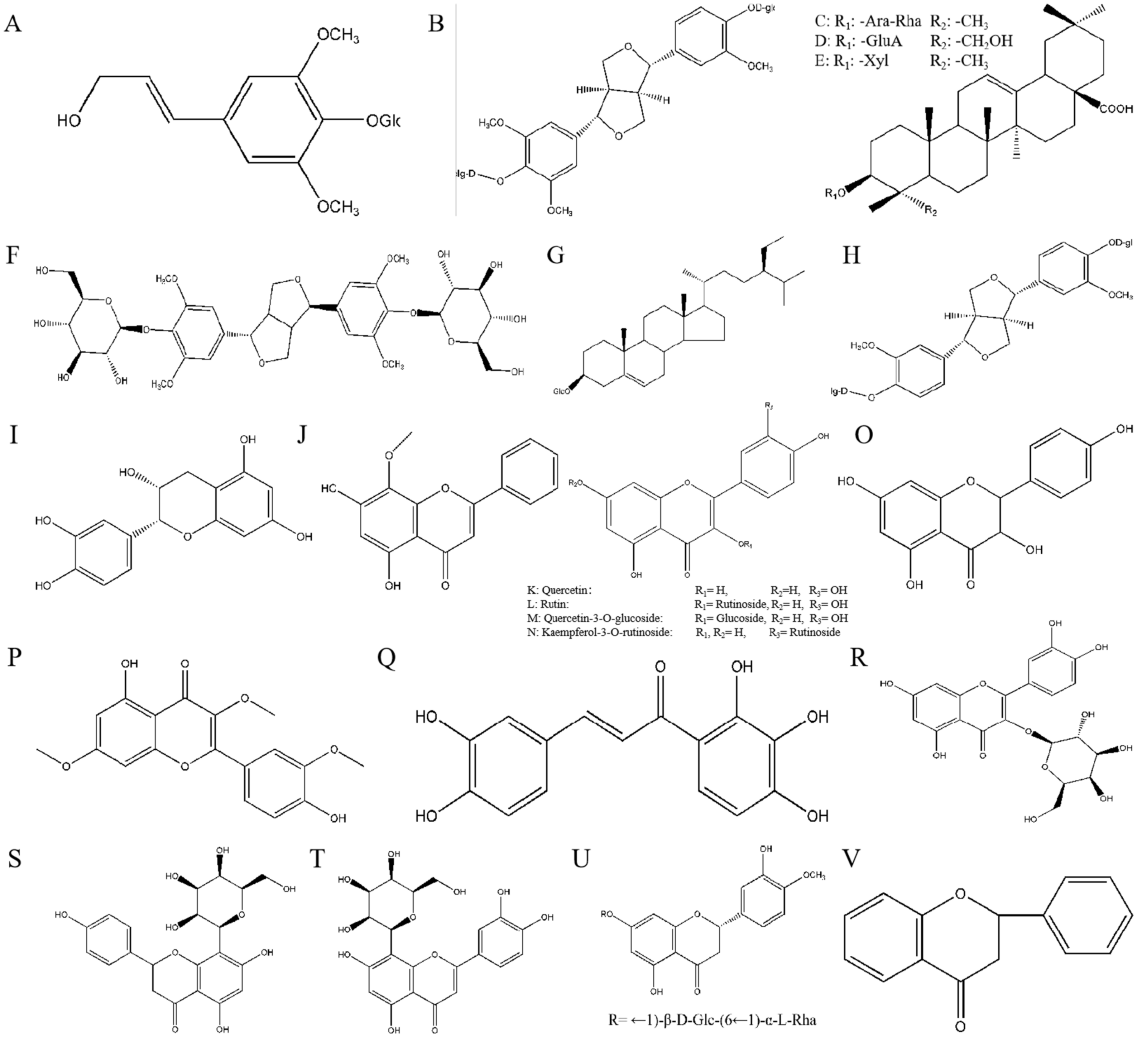
The chemical structures of eleutherosides **(A–H)**. **(A)**: Eleutheroside B, **(B)**: Eleutheroside D, **(C)**: Eleutheroside K, **(D)**: Copteroside B, **(E)**: Songoroside A, **(F)**: Eleutheroside E, **(G)**: Daucosterol, **(H)**: Pinoresinol-4,4′-di-β-O-D-glucoside. The chemical structures of flavonoids and 2-phenylchromogenon **(I–V)**. **(I)**: Epicatechin, **(J)**: Wogonin, **(K)**: Quercetin, **(L)**: Rutin, **(M)**: Quercetin-3-O-glucoside, **(N)**: Kaempferol-3-*O*-rutinoside, **(O)**: Kaempferol, **(P)**: Pachypodol, **(Q)**: Okanin, **(R)**: Hyperoside, **(S)**: Vitexin, **(T)**: Polygonin, **(U)**: Hesperidin, **(V)**: 2-phenylchromogenone.

Eleutherosides are sugar or sugar derivatives that include amino sugar and uronic acid, along with other non-sugar substances, linked through the terminal carbon atoms of the sugar molecules. The non-sugar component is aglycone or ligand, and the linked bond is aglycone. The roots and rhizomes contain a variety of eleutherosides, which can be classified as eleutheroside E, daucosterol, eleutheroside B, eleutheroside D, eleutheroside K, songoroside, copteroside B, and pinoresinol-4,4′-di-β-O-D-glucoside (Figures 3A–H) (52).

### 3.3 Flavonoids

Flavonoids are present in all AS components, with the leaves containing the highest content. The flavonoid content in leaves is affected by factors including the harvest period, producing area, storage time, and storage conditions and the highest content levels are observed in July. The flavonoid content decreases with the increasing storage time and high humidity (53).

Flavonoids are a class of yellow pigments derived from 2-phenylchromogenone (Figure 3V). Flavonoids have a C6-C3-C6 structure. Figures 3I–U shows that the main flavonoids isolated from AS include epicatechin, wogonin, quercetin, kakaferol, and rutin (54).

## 4 Extraction and purification of bioactive compounds of AS

### 4.1 Polysaccharides

With the pursuit of high-quality health, the nutritional and medicinal values of AS polysaccharides have attracted much attention. Various traditional and feasible extraction methods have been established and tested. Table 3 summarizes the extraction and purification conditions of polysaccharides in AS, as well as the yield and structure of polysaccharides from different origins. According to the extreme hydrophilicity and alcohol insolubility of AS, water is often used as a solvent or precipitant. The advantages of hot water extraction (HWE) include practical operation, simple equipment, and easy implementation. Crude polysaccharides can be directly extracted by ethanol precipitation, which is suitable for almost all water-soluble polysaccharides. This method separates the polysaccharide by reducing the dielectric constant of the aqueous solution (1). However, HWE also has significant disadvantages, including a time-consuming process, low efficiency, large solvent consumption, and high temperatures. To improve the extraction efficiency, polysaccharides have been extracted by ultrasound-, microwave-, and enzyme-assisted extraction. Previous research determined the yield of water-soluble polysaccharides from the fruit of AS in Gansu as 3.81% ± 0.18%. Compared with HWE, the yield increased by 154% (42, 43). However, a difference was observed in the molecular weight due to the damaged polysaccharide structure. This damage is attributed to the localized excessive vibration and high temperatures. The extracted crude polysaccharide of AS contains impurities such as proteins, inorganic salts, and pigments, which affect the subsequent analysis. The crude polysaccharide of AS is typically isolated and purified to remove proteins and pigments. The Sevag technique is often applied to remove proteins due to its mild conditions and weak influence on the polysaccharide structures. However, this type of purification requires repeated treatments, which affect the final yield. As a consequence, macroporous resins or dialysis bags are usually necessary to repurify crude polysaccharides (43, 44).

Chain sequencing and structural domains of carbohydrates have been the focus of much in-depth research due to the needs of pharmacology, toxicology, and structural modification. According to the order and mode of main chain glycosylation and the type of monosaccharide, the AS structure can be divided into three types, namely, RG-I, XGA, and AG-II. The RG-I type is mainly high in Rha and GALA, which are linked by 1 → 4 glycosidic bonds. Thus, most polysaccharides belong to the RG-I structure. In addition, polysaccharides with Rha/Gala ratios between 0.25 and 0.73 are easily induced to pectin. The polysaccharide extracted by Hu et al. was composed of glucose, galactose, rhamnose, arabinose, mannose, xylose, and glucuronic, with a molar ratio of 8.83:7.90:4.74:4.55:2.80:2.39:1.00, belonging to the RG-I type. The XGA structural domains represent certain polysaccharides with a high xylose content. For example, the polysaccharides extracted by Li et al. contained xylose (12, 38, 42). In addition, the XGA structural domain coexists with the RG-I structural domain in other samples of AS polysaccharides (55). Lee et al. found that AG-II type Ara and Gal are linked by a 1 → 3 glycosidic bond (48). Moreover, the purified ALP-1 extracted by Hu consists of galactose, glucose, mannose, and arabinose in a molar ratio of 6.1:2.1:1.1:1.0, with the main chain structure of 1,6-α-D-Galp residue, α-D-manp-(1 → 3)-α-L-arf residue at the O-3 position, and α-D-Galp residue at the O-4 position.

### 4.2 Eleutherosides

Solvents can be used to extract eleutherosides (Table 4). Eleutherosides have strong hydrophilicity, and water, ethanol, or methanol with a high polarity which can be used as the solvent for extraction. To enhance eleutheroside B and E yields, Na-Ri Kim et al. employed the enzyme Novozyme 33095 culture solution to treat AS (78). Enzymes can combine with pectin in the cell walls and decrease the adhesion to the active substance, consequently increasing the dissolution of eleutheroside. Following treatment with Novozyme 33095, eleutheroside B and E yields increased by 25 and 29%, respectively. The optimal extracting conditions have been reported as follows: extraction temperature of 80°C; soaking time of 5 h; ethanol volume fraction of 70%; and material-to-liquid ratio of 1:6 (60).

**Table 4:** Summary of the extraction and purification processes of eleutherosides from *Acanthopanax senticosus*.

		Extraction					Purification				
Origin	Parts	Solvent type	S/L ratio	Power (W)	Temperature (°C)	Time (h)	Elution type	Flow rate	Resinous type	Content	
Jilin, China	Fruit	70% ethanol	1: 20	Ultrasound:450	–	0.8	–	–	–	3.57 mg/g	(56)
Heilongjiang, China	–	Ionic liquid	1: 25	Ultrasound:250	–	0.5	–	–	–	Eleutherosides B: 3.3%, eleutherosides E: 4.6%	(57)
Heilongjiang, China	Roots, rhizomes	Surfactant	1: 20	Ultrasound:250	50	0.7	–	–	–	Eleutherosides B: 1.06 ± 0.04 mg/g, eleutherosides E: 2.65 ± 0.12 mg/g	(17)
Zhejiang, China	Stem	Tea saponin	1: 20	Ultrasound:250	50	0.7	Chloroform:methanol:isopropanol:water: 5:6:1:4	1 mL/min	High speed countercurrent chromatograph: TBE300B	Eleutherosides B 1.18 ± 0.05 mg/g, eleutherosides E 2.87 ± 0.12 mg/g	(58)
Heilongjiang, China	Stem	Betaine:triethanolamine:magnesium chloride hexahydrate: 1:4:0.08	1: 14	Microwave:600 ultrasound: 500	59	0.7	95% ethanol	6 BV/h	NKA-9, D101, HPD-400, ADS-17, AB-8	Eleutherosides B 0.16 mg/g, eleutherosides E 0.04 mg/g	(59)
Hebei, China	Leaf	70% ethanol	1: 6	–	80	5	60% ethanol	27.22 × 10^−6^ m^3^/min	AB-8	Saponins: 44.1%	(60)
Heilongjiang, China	Root, stem	–	–	–	–	–	30% ethanol	1 BV/h	AB-8	Eleutherosides B: 7.95%, eleutherosides E: 11.15%	(61)
Heilongjiang, China	–	Water	1: 6	–	–	0.5	60% ethanol	3 BV/h	HPD100C	Eleutherosides B: 0.28 mg/g, eleutherosides E: 1.49 mg/g	(62)
Heilongjiang, China	Stem	Choline chloride: fructose: 1:1	1: 20	–	80	2	–	–	–	eleutheroside E: 1.96 mg/g	(63)
Heilongjiang, China	Rhizome	Choline chloride: ethylene glycol: 1: 3	1: 10	Microwave: 400	75	0.2	–	–	AB-8	eleutheroside B: 0.282 mg/g, eleutheroside E: 1.486 mg/g	(64)
–	Root, stem	70% ethanol	1: 10	–	–	1	30% ethanol	3. 5 BV/h	D101	18. 35%	(65)
Heilongjiang, China	Leaf	Water	–	–	–	2	–	–	–	–	(66)
Liaoning, China	–	65% ethanol	–	–	–	–	–	–	–	eleutheroside E_2_ 1.3 mg/kg, tachioside 0.53 mg/kg, carotenoside 2.01 mg/kg	(67)
Jilin, China	Fruit	Water	1: 5	–	–	–	Chloroform-methanol: 10∶1 ~ 1∶1	–	–	Acanthopanthoside A 41 mg/kg, Acanthopanthoside B 40 mg/kg	(68)
–	–	Methanol	–	–	–	0.5	–	–	–	Syringin: 3.5 mg/kg, Acanthopanthoside E: 4 mg/kg, cirtrusin A: 2 mg/kg, cirtrusin B: 1 mg/kg, syringophenol glucoside: 0.85 mg/kg	(69)
–	Stem	Water	–	–	–	–	Dichloromethane-methanol	–	–	Syrigin: 0.02 mg/g, eleutheroside B_1_: 0.02 mg/g, eleutheroside D: 0.02 mg/g	(70)
Liaoning, China	Stem	65% ethanol	–	–	–	2	30% ethanol	–	–	Eleutheroside B_2_: 0.17 mg/g	(71)
Gongju, South Korea	Fruit	Methanol	1: 3	–	–	–	Chloroform, ethyl acetate, n-butanol	–	–	Carotenin, eleutheroside K, pinechinoside A, Cephaloside B	(72)
Jilin, China	Stem	Water	–	–	–	–	CHCI_3_, ethyl acetate, n-butanol	–	–	Eleutheroside B, eleutheroside E	(73)
National Institutes for Food and Drug Control	–	80% ethanol	1: 10	–	–	–	–	–	–	Eleutheroside B: 7.63%	(74)
Sichuan, China	Stem, root bark	Water	–	–	–	–	Water, 15, 25, 75% ethanol	–	D101	Eleutheroside E: 0.19–2.86 mg/g	(75)
Heilongjiang, China	Root, rhizome	70% ethanol	–	–	–	–	Water, 10, 30, 50, 95% ethanol	–	D101	Eleutheroside D: 0.05 mg/g, Pinoresinol-4, 40-diethyl-O-D-glucoside: 0.09 mg/g, Eleutheroside E: 0.25 mg/g, 5-methoxylariciresinol-4-O-b-D-glucopyranoside: 0.04 mg/g, Eleutheroside B: 0.13 mg/g, Eugenol-4-o-b-d-glucoside: 0.15 mg/g, Eleutheroside E_2_: 0.06 mg/g	(76)
Beijing, China	–	80% ethanol	–	–	–	–	Water, 20, 50, 90% ethanol	–	–	Eleutheroside B: 7.45%	(77)

The development and utilization of green solvent systems have recently become a hot spot. In 2003, researchers reported a new type of green solvent denoted as the deep eutectic solvent (DES) (79). DES consists of a hydrogen bond acceptor (HBA) and a hydrogen bond donor (HBD). The HBAs are mostly quaternary ammonium salts and zwitterions, while the HBDs are carboxylic acids, amides, and polyols. The HBD is the main factor affecting the polarity of DES, and the initial polarity of DES is similar to that of 70% of ethanol. DESs are non-toxic and produce minimal environmental pollution. They have high solvency, high extraction efficiency, high selectivity, and a tunable nature compared with traditional organic solvents. The extraction rate of isofraxidin by DES (choline chloride and citric acid) has been reported as 1.56 mg/g, which is 2–3 times that of conventional solvents (80). Ultrasound- and microwave-assisted extraction methods have been employed to improve the movement frequency, macromolecule speed, and extraction efficiency. Du et al. applied alternative ultrasonic- and microwave-assisted methods to extract isofraxidin (81). This method avoids the decomposition and isomerization of natural products due to the long extraction time. Eleutheroside E has also been extracted by ultrasonic extraction mass spectrometry, with DES composed of choline chloride and fructose (1:1) as the extraction solvent (63). The content of glycoside E reached 1.96 mg/g, which was 5–6 times higher than that of 70% of ethanol. Previous research combined microwave ultrasound-assisted extraction with 3-element DES (betaine, triethanolamine, and MgCl_2_·6H_2_O) to extract eleutherosides. The yields of isofraxidin, eleutheroside E, eleutheroside B, protocatechuic acid, chlorogenic acid, and ursolic acid reached 18.76, 49.38, 174.82, 2.98, 457.39, and 16.62 μg/g, respectively, representing a 1.04- to 3.19-fold increase compared to those obtained through conventional extraction methods (59).

Eleutherosides in crude extract often coexist with other active ingredients. The separation and purification of eleutherosides are generally carried through ethanol precipitation. High-purity eleutheroside B, eleutheroside E, and isofraxidin can be obtained using a combination of ionic liquid-loaded (1-butyl-3-methyl-imidazolium bisulfate) AB-8 macroporous resin and DESs with molecularly imprinted separation technology (64). Table 4 shows the extraction and purification conditions.

### 4.3 Flavonoids

The extraction methods of total flavonoids include reflux extraction, ultrasonic/microwave extraction, supercritical fluid extraction, and rapid solvent extraction (Table 5), with ethanol, methanol, enzyme, and DESs typically selected as the solvents. Compared with traditional solvent extraction, the extraction rate of supercritical CO_2_ extraction is reported to increase by 33.26% (98). This technology is safe, environmentally friendly, has no reagent residue, and consumes little energy. Due to the expensive supercritical extraction equipment and other factors, the ethanol–water reflux extraction of total flavonoids is more suitable for large-scale production. Liu et al. employed enzyme-assisted ultrasonication to extract total flavonoids (85), determining the optimal extraction process as follows: cellulase to pectinase ratio of 3:2; addition of 6,960 U/g of enzymes; enzyme treatment time of 59.80 min; temperature of 53.70°C; and pH of 6.05. Based on these settings, the total flavonoid yield was 36.95 ± 0.05 mg/g.

**Table 5:** Summary of the extraction and purification processes of flavonoids from *Acanthopanax senticosus*.

Origin	Parts	Extraction					Purification			Content	
		Solvent type	S/L ratio	Power	Temperature (°C)	Time (h)	Elution type	Flow rate	Resinous type		
–	Root	60% ethanol, supercritical CO_2_	–	Supercritical CO_2_ extract_:_ 40 Mpa	78/55	2–3	60% ethanol	2 mL/min	NKA-9	Flavonoids: 58.1%	(82)
–	–	55% ethanol	1: 45	Ultrasonic: 300 W	72	1.2	60% ethanol	3 BV/h	HPD-600	Flavonoids: 50.57%	(83)
Heilongjiang, China	Leaf	100% methanol	1: 15	100 Pa	100	0.1	–	–	–	–	(84)
–	–	Ellulase:pectinase: 3:2	1: 20	Ultrasonic: 300 W	55	0.9	–	–	–	Flavonoids: 36.95 ± 0.05 mg/g	(85)
–	–	Glycerol:levulinic acid: 1:1	1: 18	Ultrasonic: 500 W	55	1.2	–	–	AB-8	23.93 ± 0.07 mg/g	(53)
–	–	60% ethanol	1:40	–	60	1.1	60% ethanol	3 BV/h	HPD-600	Flavonoids: 76.4% ± 2.1%	(86)
–	–	75% ethanol	–	–	70	2.5	–	–	–	–	(3)
Hunan, China	–	95% ethanol	1: 5	–	–	3	50% ethanol	5 mL/min	D101	Flavonoids: 30.13%	(87)
–	–	55% ethanol	1: 45	–	72	1.2	–	–	–	Flavonoids: 24.11 ± 0.17 mg/g	(88)
Jilin, China	Leaf	Supercritical CO_2_	1: 1.6	32 Mpa	45	3	–	–	–	–	(89)
Gansu, China	Stem, leaf	70% ethanol	1: 35	600 W	60	0.6	–	–	–	Flavonoids: 28.11 ± 0.19 mg RE/g	(90)
Jilin, China	Leaf	70% ethanol	–	Microwave: 500 W	Ultrasonic: 60	Microwave: 0.02 Ultrasonic: 0.5	–	–	–	Rutin: ultrasound: 0.39 mg/g Microwave: 0.38 mg/g	(91)
Jilin, China	Leaf	60% ethanol	–	–	–	–	60% ethanol	20 mL/ min	–	Flavonoids: 60%	(92)
Hubei, China, Seoul, Korea	Leaf	70% methanol	–	–	–	–	–	–	–	Rutin: 0.07–19.33 mg/g, quercetin-3-*O*-glucoside: 0.06–5.30 mg/g, kaempferol-3-*O*-rutinoside: 0.08–5.03 mg/g	(93)
–	–	60% methanol	1: 20	–	–	2	–	–	–	Flavonoids: 3.02–137.42 mg/g	(94)
Gansu, China	Leaf	50% ethanol	–	Microwave: 300 W	–	0.13	–	–	–	Quercetin: 0.86 mg/g, quercitrin: 1.65 mg/g, rutin: 2.19 mg/g, hyperin: 2.68 mg/g	(95)
Heilongjiang, China	–	50% ethanol	1: 50	–	50	Microwave: 0.15	–	–	–	–	(96)
Jilin, China	Leaf	50% ethanol	1: 20	–	–	1.5	–	–	–	Flavonoids: 51.92 mg/g	(97)

Microporous adsorbent resin can be applied to separate the chemical components of herbal medicines. This technique has the advantages of fast adsorption, large adsorption capacity, high elution rate, and simple resin regeneration. Commonly used eluents are water, methanol, ethanol, acetone, and ethyl acetate. Wang et al. employed HPD-600 resin to increase the purity of flavonoids to 50.57% (86). The adsorption process was carried out following quasi-secondary kinetics, in accordance with the Freundlich adsorption model.

## 5 Biological activities

### 5.1 Anti-fatigue properties

Fatigue can be divided into sports fatigue and mental fatigue according to its attributes. The former occurs mostly in people who engage in high-intensity physical labor for a long period, while the latter denotes the excessive use of the brain (99). Sports fatigue will lead to mental instability and an accelerated heartbeat with lactic acid accumulation, while central fatigue will produce side effects such as memory loss, drowsiness, and emotional instability (100). Increased exercise endurance is the most potent macroscopic form of fatigue resistance (101).

**Figure 4:**
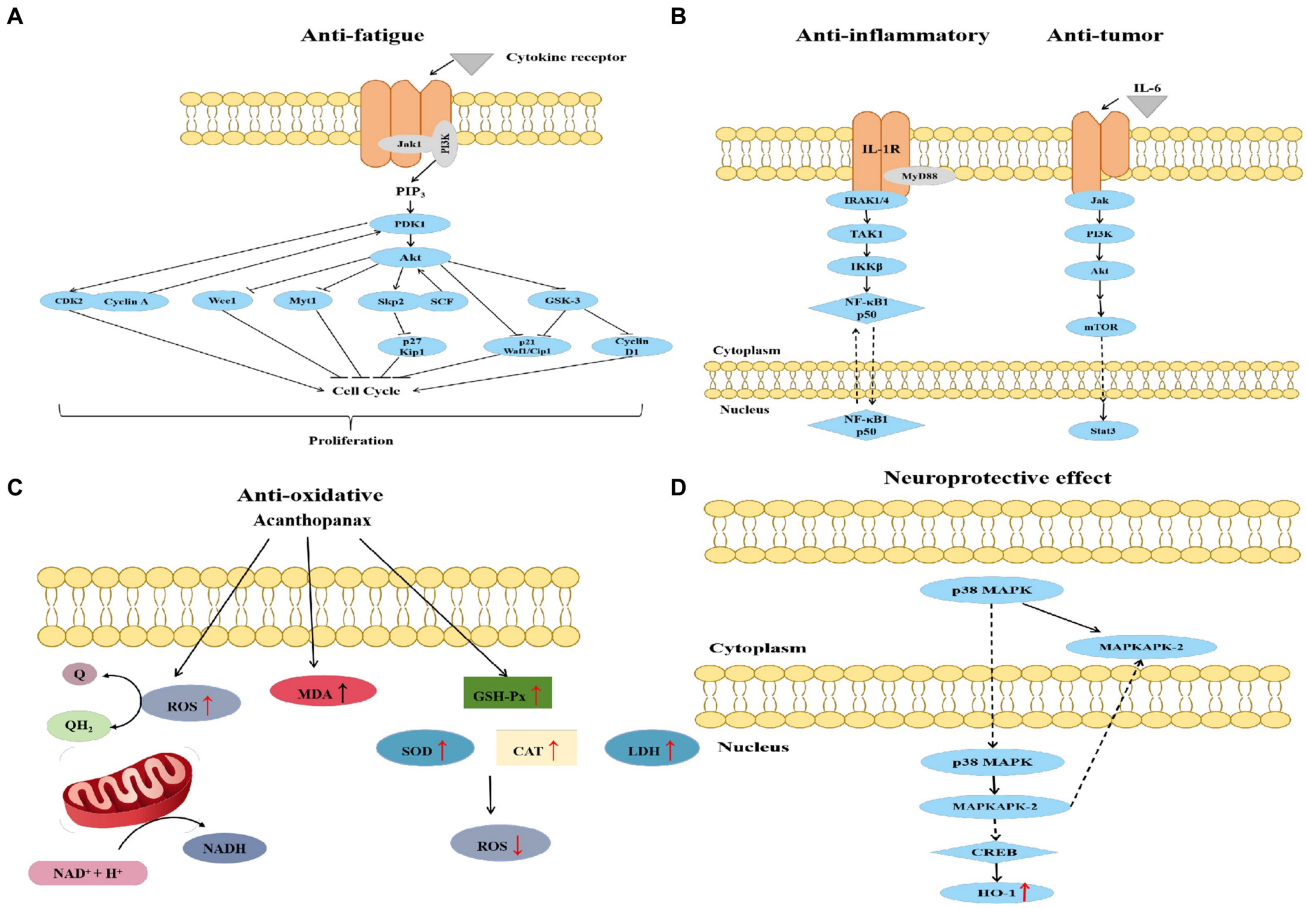
Schematic diagram of anti-fatigue **(A)**, anti-inflammatory, anti-tumor **(B)**, anti-oxidative **(C)**, and neuroprotective **(D)** mechanisms of *Acanthopanax senticosus*.

Studies have reported the anti-fatigue effects of the AS extract (102). Fatigued mice fed with the AS extract showed increased weight-bearing swimming time, tissue glycogen content, and learning memory ability, and decreased blood lactate and urea nitrogen contents. Gao et al. constructed a network diagram of “active ingredients–anti-fatigue targets” to screen the critical components of AS, including eleutherosides B, B_1_, C, and E (103). The core anti-fatigue targets were predicted to be HSP90AA1, STAT3, VEGFA, and MMP9. Molecular docking predicted that both core components and key targets could spontaneously bind, and the binding ability was similar to that of the clinical drug ATP-2Na. Furthermore, the binding activities of eleutherosides B and B_1_ were higher than those of the key proteins. The anti-fatigue effect of eleutherosides is mainly executed by regulating HIF-1, PI3K-Akt, insulin signaling pathway, and substance metabolism through biological processes such as drug responses and the positive regulation of cell proliferation (Figure 4A) (52). Cheng et al. established fatigue mouse models to confirm that Erythrina saponins E can reduce blood urea nitrogen (BUN) and enhance the activity of serum lactate dehydrogenase (LDH) to prolong the exhaustion swimming time of mice (104). BUN, the end product of protein metabolism, is an important blood biochemical indicator related to fatigue. Eleutheroside has the ability to reduce the formation of BUN after exercise. Eleutheroside can increase the activity of serum LDH, accelerate the body’s ability to clear lactic acid, and play a role in delaying fatigue. In addition, eleutheroside B regulates the expression of factors associated with the Keap1/Nrf2/ARE signaling pathway to improve learning memory (105).

### 5.2 Anti-inflammatory properties

Fan, Kim, Zhang et al. demonstrated that AS has protective and ameliorative effects on the gastrointestinal tract (106–108). Polysaccharides prevent the lipopolysaccharide-induced amplification of inflammatory mediators via a decrease in ileal mRNA abundance of TNF-α, inducible NO synthase, and IL-1β concentrations. Protein expression of hypoxia-inducible factor-1α, cyclooxygenase-2, and κ-B p65 is also reduced in ileal tissues. Moreover, the height of the ileal villi and the appearance of the epithelial villi of piglets fed with AS polysaccharides have been reported to improve, and polysaccharides can alleviate the reduced average daily feed intake of piglets and decrease the incidence of diarrhea and diarrhea index (106). The extrusion of AS leaves increases the protection of the gastric mucosa and prevents C48/80-induced acute gastric mucosal injury (107). Previous research has shown that serum concentrations of 5-hydroxytryptamine and histamine were reduced in C48/80-modeled rats, while the expression of Bax and Bcl-2 was improved. A similar effect was observed for 200 mg/kg of AS on famotidine, a histamine H2 receptor antagonist, and normalized mucus secretion. These polysaccharides significantly increased the survival of flies and reduced the proliferation and differentiation of intestinal stem cells in response to dextran sodium sulfate through the epidermal growth factor receptor, Jun-N-terminal kinase, and Notch signaling pathways (108). Sodium dodecyl sulfate can induce the melanin phenotype and disrupt epithelial renewal. Therefore, polysaccharides are also effective in reducing sodium dodecyl sulfate-induced epithelial cell death and the accumulation of reactive oxygen species and antimicrobial peptides. AS polysaccharides also have antioxidant effects and improve the level of oxidative stress induced by cerebral ischemia–reperfusion injury (109).

Isofraxidin in AS attenuates the inflammatory response in the lung tissue by decreasing the levels of tumor necrosis factor-α, interleukin-6, neutrophils, and NF-kB (110). Myeloperoxidase activity is also reported to be significantly reduced in the lung tissue. The protective effect on the lungs is associated with the inhibition of cyclooxygenase-2 protein expression. Eleutherosides E can effectively inhibit high-altitude heart injury by regulating NLRP3 inflammatory vesicle activation and cellular death in cardiac tissues (111). Song et al. applied *Edwardsiella ictaluri* to reveal that AS extract significantly reduced the expression of proinflammatory cytokines including IL-1 (Figure 4B) (112). *Edwardsiella ictaluri* infection increased IL-1 expression and activated the NF-кB/MyD88 pathway. The results showed that the expression of the NF-кB/MyD88 pathway significantly decreased.

### 5.3 Antioxidant properties

The excessive production of reactive oxygen species induces the development of cardiovascular diseases such as atherosclerosis and hypertension. It has been shown that AS can reduce lipid peroxidation, with an increase in the activity of superoxide dismutase and catalase in cells (Figure 4C) (113). Oxidative stress also contributes to the development of diabetic complications. AS polysaccharide can significantly reduce the levels of lipid peroxidation markers, such as thiobarbituric acid-responsive substances and lipid hydroperoxides (114). The activities of enzymatic antioxidants, superoxide dismutase, and catalase also significantly increased. Wang et al. showed that AS extracts protected against oxidative stress by increasing the activities of antioxidant enzymes and GSH/GSSG ratios in serum and liver homogenates (115). Both medium and high doses of AS aqueous extracts significantly increased Nrf2 and antioxidant enzymes. The antioxidant properties of AS polysaccharides have been reported as equally effective in zebrafish *in vivo*. The purified polysaccharide can significantly scavenge hydrogen peroxide and reduce hydrogen peroxide-induced cell death in zebrafish (116).

### 5.4 Antitumor and anticancer properties

AS has good antitumor and anticancer pharmacological effects. Cytotoxicity experiments revealed that the cells of colon (SW 620), breast (MDA-MB-231, MCF-7), gastric (Kato-III), ductal (BT474), bronchial (Chago K-1), prostate (PC-3), and hepatic hepatoblastoma (Hep-G2) cancers produced certain toxic responses and inhibitory effects under the action of AS (117–121). Syringin from AS can inhibit the proliferation and migration of breast cancer cells and promote apoptosis by regulating the EGFR-RAS–RAF–MEK–ERK and PI3K-AKTCOX-2 signaling pathways (Figure 4B) (117). The ethanol extract of AS induced liver cancer cell apoptosis by inhibiting nuclear factor-κB activity, with an inhibition rate of 57.55% in HepG2 cells (121). The extract can also decrease the protein levels of matrix metalloproteinase-2 (MMP-2), MMP-9, t-protein kinase B, and p-Akt and increase the protein level of E-cadherin. The results demonstrated that the number of cells penetrating the basement membrane was significantly reduced after the treatment with the extract of AS, which suggested that the extract can inhibit the invasion of HepG2 cells. Yamazaki et al. found that isofraxidin, a coumarin component from AS, significantly inhibited hepatocellular carcinoma cell invasion (120). Isofraxidin inhibits terephthalic acid-induced matrix MMP-7 expression in hepatocellular carcinoma cells by preventing ERK1/2 phosphorylation. The IC_50_ values of the extract for Kato-III and SW 620 cells have been determined as 72.9 and 73.4 μg/mL, respectively (118). Glycoprotein in AS can significantly suppress the metastasis of colon cancer 26-M3.1 cells in the lung (122). In particular, glycoprotein activates macrophages to produce various cytokines and inhibit tumor metastasis or enhance host natural immunity (123).

### 5.5 Neuroprotective effects

AS can protect neurons and thus has important applications in drug development for Alzheimer’s and Parkinson’s diseases (PD). Mice treated with AS have shown a reduction in climbing time, an increase in the number of voluntary movements, and a reduction in cognitive and spatial memory deficits (28, 124). Synaptic nucleoprotein is the key factor in the pathogenesis of PD (125). In a previous study, after the WT-Syn and A53T-Syn transgenic cells were treated with the extract, the levels of synapsin, caspase-3, parkin, and other related proteins returned to near-normal levels (126). This is consistent with the results reported by Liu et al. (127). The neuroprotective effect of AS can be mediated by the activation of parkin and HO-1 expression, which inhibits caspase-3 activity and concomitantly reduces nitric oxide/ROS production, thereby inhibiting neuronal apoptosis. HO-1, an inducible enzyme present in most cell lines, is protective against glutamate-induced neuronal cell death (128). AS induces HO-1 expression mainly through the p38-CREB pathway (Figure 4D). Previous studies has shown that AS may regulate the structural composition of the gut microbiota, improve metabolic disorders, reduce inflammatory factors, and reverse the PD process (28). Lu et al. found a significant increase in Firmicutes and a significant decrease in Actinobacteria expression in the intestine of ASH-treated Parkinson mice (28). Short-chain fatty acids produced by the fermentation of Firmicutes promoted metabolism by acting on G protein-coupled receptors (129). Actinobacteria cause damage to dopaminergic neurons by producing protease inhibitors (130). These conclusions highlight a novel mechanism underlying the effects of AS on the “brain–gut axis.”

## 6 Applications

### 6.1 Functional food

AS is used in wine, beverages, fruit cakes, tea drinks, yogurt, and other products. Zhang et al. employed the rhizome as a raw material to prepare a beverage with the characteristic tea fragrance (131). Zhao et al. also prepared health-preserving tea, and its raw ingredients were AS berries and longan (132). The formula of the health-preserving tea is the ratio of AS to longan was 1:4, with 4% sugar and 0.1% citric acid. Ma et al. produced nutritional yogurt using fresh leaves and milk as the main raw materials. The formulation included 0.2% of xanthan gum, 0.3% of sodium carboxymethyl cellulose, 20% of fresh juice, 7% of sugar, and 3% of milk powder (133). Moreover, Niu et al. developed AS yogurt (134) and He et al. applied AS berries as raw materials to produce a fruit cake. The optimum production formula consisted of sucrose (37.9%), citric acid (2.95%), xanthan gum (2.1%), sodium alginate (2.1%), and agar (1.58%) (135).

### 6.2 Medicine

AS is a traditional medicinal plant in China. Numerous medicines have been developed with AS raw materials, such as injections, tablets, granules, and capsules. Chinese researchers have developed AS dispersible tablets characterized by a reasonable prescription, a feasible process, a high dissolution degree, and good dispersion uniformity (136). Such medicines can treat weakness, appetite loss, cough, asthma, insomnia, and cardiovascular and cerebrovascular diseases (137). Studies have shown that AS delays physical fatigue and relieves mental fatigue by improving memory retention and increasing spontaneous activity (102). AS is widely used in clinical practice to treat transient ischemic attacks, and its injection is a Chinese patent medicine often used for treating ischemic heart disease.

### 6.3 Animal dietary additive

Early weaning of piglets leads to decreased growth performance and an increased incidence of diarrhea and disease. Antibiotics are traditionally used to prevent and treat intestinal disorders caused by weaning stress. However, the continued use of antibiotics results in drug and antibiotic residues in the animals. Novel feed additives are thus required to replace antibiotics. AS extract, as a promising alternative to antimicrobial agents, can effectively enhance the digestibility and absorption of amino acids in weaned piglets (138). Yin et al. found that AS promoted the diversity of cecal microbiota, with the number of lactic acid bacteria increasing (*p* < 0.05) and *Escherichia coli* decreasing (*p* < 0.05) as the weaning age progressed.

In summary, the application of AS is mainly centered on functional food and medicine. Despite the great progress made by recent research, the application of AS is still associated with several shortcomings. For example, the development of the product has remained in the laboratory stage and cannot be well industrialized. This indicates that the research on AS lacks depth. Moreover, the product category is simple, which cannot meet the demand of the consumers for healthy lives. The lack of standards for the testing results in the uneven quality of AS products on the market. In view of the aforementioned problems, scholars should focus on the transfer of experiments from the small scale to the industrial scale to achieve industrialization. Additional AS products should also be developed such that consumers can gradually accept them and improve their living standards. Furthermore, scholars and experts should formulate corresponding production and inspection standards to improve the quality of such products and increase consumer satisfaction.

## 7 Conclusion

AS is rich in active ingredients, has a wide range of pharmacological effects, and shows great potential for clinical applications. However, controversies exist in some areas related to whether AS belongs to the category of medicine-food homology. Moreover, while emerging extraction technologies can improve efficiency, they may decrease the yield, resulting in a greater waste of resources. Although DES extraction is environmentally friendly, the solvent recovery of DES is difficult and reduces extraction efficiency. In terms of application, AS-related health products are limited. There is also a lack of quality control standards for AS products. Furthermore, in recent years, new delivery systems using biological macromolecules as wall materials are developing rapidly, providing humans with new directions and technologies in food and medicine industries. AS and its active components can be applied as a wall core, while biological macromolecules can be adopted as the wall material to construct a drug delivery system. However, the application of AS in this field is relatively new and requires much work. Thus, further research should focus on the following: (1) employing optimized extraction methods to improve the yield of bioactive compounds in AS; (2) establishing a clear definition of AS and formulating relevant quality control indicators; (3) conducting more clinical trials to verify the health benefits of AS for humans; (4) exploring the application of AS in the prevention and treatment of diseases; (5) developing healthcare foods and other products based on AS.

In conclusion, with the development of science and life, health has garnered increasing attention. AS is rich in nutrients and holds economic and developmental value. Moreover, the promotion of AS can maintain, innovate, and develop traditional Chinese medicine culture and products. With the further understanding of AS and the improvement of technology, AS will soon be widely employed in both clinical and functional food sectors.

## Author contributions

XZ: Software, Writing – original draft, Writing – review & editing. LG: Software, Writing – original draft, Writing – review & editing. LZ: Software, Writing – review & editing. KW: Software, Writing – review & editing. YG: Writing – review & editing. JL: Writing – review & editing. SY: Writing – review & editing. NJ: Writing – review & editing. YZ: Supervision, Writing – review & editing. XY: Writing – review & editing. BL: Supervision, Writing – review & editing.
